# Narrowing the gap between research and policy: using rapid evaluation during the COVID-19 crisis

**DOI:** 10.1177/17579139221138449

**Published:** 2023-01-24

**Authors:** K Anil, D Watson, J Alagil, R Dewar-Haggart, S Fearn, C McGrath, C Meagher, S Muir, M Barker

**Affiliations:** School of Health Professions, Faculty of Health and Human Sciences, University of Plymouth, Peninsula Allied Health Centre, Derriford Road, Plymouth PL6 8BH, UK; Global Health Research Institute, School of Human Development and Health, Faculty of Medicine, University of Southampton, UK; School of Health Sciences, Faculty of Life and Environmental Sciences, University of Southampton, UK; College of Applied Medical Sciences, Health Rehabilitation Department, King Saud University, Kingdom of Saudi Arabia; School of Primary Care, Population Sciences and Medical Education, Faculty of Medicine, University of Southampton, UK; School of Human Development and Health, Faculty of Medicine, University of Southampton, UK; NIHR Southampton Biomedical Research Centre, University of Southampton and University Hospitals Southampton NHS Foundation Trust, UK; MRC Lifecourse Epidemiology Unit, University of Southampton, UK; School of Health Sciences, Faculty of Life and Environmental Sciences, University of Southampton, UK; MRC Lifecourse Epidemiology Unit, University of Southampton, UK; School of Health Sciences, Faculty of Life and Environmental Sciences, University of Southampton, UK; NIHR Southampton Biomedical Research Centre, University of Southampton and University Hospitals Southampton NHS Foundation Trust, UK; MRC Lifecourse Epidemiology Unit, University of Southampton, UK

## Introduction

An evidence base is vital to ensure well-informed policies with systematic processes.^1,2^ However, A research-to-policy gap exists that is widened by the discrepancy between the extensive time it takes to conduct conventional academic research and the short timescale over which policy-makers are often required to make decisions.^2^ This is especially concerning in times of crisis, such as the COVID-19 pandemic, that demanded an avalanche of data, analysis, and interpretation to be provided over a very short period of time. Rapid evaluation can be used to generate research-based evidence under pressure to inform decision-making and policy. Existing literature clearly details the steps involved in conducting rapid evaluation^3^; yet this literature contains little practical knowledge about how best to carry out such research. In this article, we reflect upon the practical implementation of rapid evaluation for an urgent project during the crisis of COVID-19.^4^

## Brief Project Background and Reflection Process

Between June and October 2020, a COVID-19 Saliva Testing Programme was piloted, where findings were reported to the UK Department of Health and Social Care (DHSC).^5^ A rapid evaluation was conducted with participants of the testing programme to generate insights that would inform the testing’s design and modification, and the next phase of future mass-testing to the UK DHSC.^4^ For further details on the project, see our published paper.^4^ We used Gibb’s reflective cycle^6,7^ as a foundation for the team to reflect on their experiences of working on a rapid qualitative project. The main ‘lessons learned’ are explained below, followed by actionable suggestions in Table 1.

**Table 1:** 

Actionable points based on our reflection categories		
Category	Specific actions for a leader	Specific actions for a team member
*The Value of a Shared Vision*	Clearly communicate the project’s goals and its importance at the start and throughout the project to maintain team motivation	Ensure you know why the project is needed and be aware of project goals from the beginning
	Plan the project in-depth as early as possible; this includes the following: • Preparing a timeline for each step of the project • Deciding deadlines • Building the team and identifying individual member’s strengths • Deciding task delegation to the team based on skill	Familiarise yourself with project timelines and deadlines
	Create regular meeting spaces with team members to communicate the following: • Project goals • Deadlines • Update on project outputs and next steps	Ensure you know how to access project information and learn to use any required project-related technology
	Consider using a platform (such as Microsoft Teams) as a central location to store and share real-time project information and documents with the team	
	Following each meeting, send a summary and action points to all team members	
*Supportive Leadership Style*	Identify skill gaps among your team and provide support/training to reduce those gaps	Familiarise yourself with your team members’ skills to identify opportunities to assist your own work and/or improve your own skills
	Listen to the opinions of your team and allow for the potential for their opinions to influence the project plans	Listen to the opinions of other team members and communicate challenges to facilitate collaborative problem solving
	Be prepared to listen to opposing opinions and make final decisions on any differences; this will be made easier by being clear on your project goals	
	Approach your senior colleagues for support and advice when needed	
	Recognise and show appreciation of team members for their efforts by providing positive feedback	Offer support to other team members where needed and if you have the capacity
	Reflect on your leadership skills, recognising your concerns and identifying ways to overcome them; use strategies such as Gibb’s Reflective Cycle for reflection	Noting the leadership skills of your project lead may help you vicariously develop your own leadership skills; look out for the following to help you: • Methods used to communicate information • Storage of information • How they use technology to support the project • How they interact with team members and the type of support they offer
*Access to Rapid Evaluation Methods and Digital Technology*	Understand the principles of a rapid approach and share them with the team at the beginning of the project to set expectations	Familiarise yourself with the principles of a rapid approach and be prepared to meet its expectations
	Clearly communicate deadlines and expectations of work timescales to your team at the start; do this by: • Storing deadlines and timelines on a central storage system that is easily accessible • Ensuring every team member knows where and how to access this information • Emphasising task expectations by outlining specific task details and workload involved • Setting clear expectations of the work schedule and arranging working hours based on your team’s availability	Ensure you are prepared to reply to communications from your project lead and team members quickly to ensure task deadlines are met
	Balance your rapid project with your other work commitments by setting specific hours and days to work on specific projects	Communicate your specific working hours to your project lead to set communication expectations
	Update project information as soon as possible and communicate these updates to your team; using a real-time reporting system (e.g. on Microsoft Teams) will greatly assist this communication	
	Create a plan that can be flexible, based on your deadlines, your team’s working hours, and skills of team members	

## Creating an Effective Team for Rapid Outputs

The team identified four main lessons critical to the success of our rapid evaluation project during a crisis: (1) the shared vision for the project held by the team, (2) effective project management, (3) the supportive leadership style, and (4) access to rapid evaluation methods and digital technology.

### The value of a shared vision

The pandemic crisis gave the work an urgency and significance felt by the team, resulting in both a personal and professional commitment to the project. Team members felt positive about the contribution they were making to the national campaign to reduce spread of the infection. This drive facilitated a proactive and supportive work culture, where team members responded to communications quickly and worked collectively to solve problems. All team members listened and valued opinions from other members and were quick to take up responsibilities if others did not have the capacity. A sense of trust and confidence were quickly built among the team members, who easily relied on each other to complete tasks and receive advice. The culture enabled the project to collect data beyond its target and produce outputs that were valued by DHSC for their timeliness and insights.

### Effective project management

Effective project management and accessible information sharing and communication were key to generating project insights within the tight deadlines required by the stakeholders. The team leader communicated the project’s goals and objectives at the beginning of the project, specifying how the project was to be completed. She also held weekly meetings to discuss experiences or challenges and real-time digital summaries accessible to all on Microsoft (MS) Teams. The well-organised communication of the project ensured all team members were up-to-date on the project and knew its immediate next steps. The team also had immediate access to all project-related documents and data via MS Teams, such as project protocols, task delegation spreadsheets, deadlines, and result summaries.

### The supportive leadership style

The team leader initially identified the strengths, skills, backgrounds, and experience of team members in order to delegate tasks accordingly. The team leader’s in-depth knowledge of her team led to inherent confidence in each member’s ability to complete the project’s tasks on time. The team consisted of 12 researchers from varied backgrounds and seniority that required active management from the team leader by listening to their opinions and concerns, and making members feel valued. Some team members were inexperienced with rapid evaluation methods and were initially worried about their contribution to the project. The team leader reassured them by stating why they were asked to be on the team and provided appropriate support where needed. When team members had opposing perspectives on aspects of the project, the team leader made the final decision through team discussion – a process made easy due to the trust built over the course of the project.

Encouraging team members to actively participate and support each other eased the management burden of a large team for the team leader, which prevented her feeling overwhelmed with project speed and enabled her instead to enjoy the process. The team leader was open about gaps in her knowledge and sought help when needed. These leadership skills were respected by the team, who felt they vicariously learned about effective leadership and believed they would be employing these skills in future team management activities.

**Figure 1:**
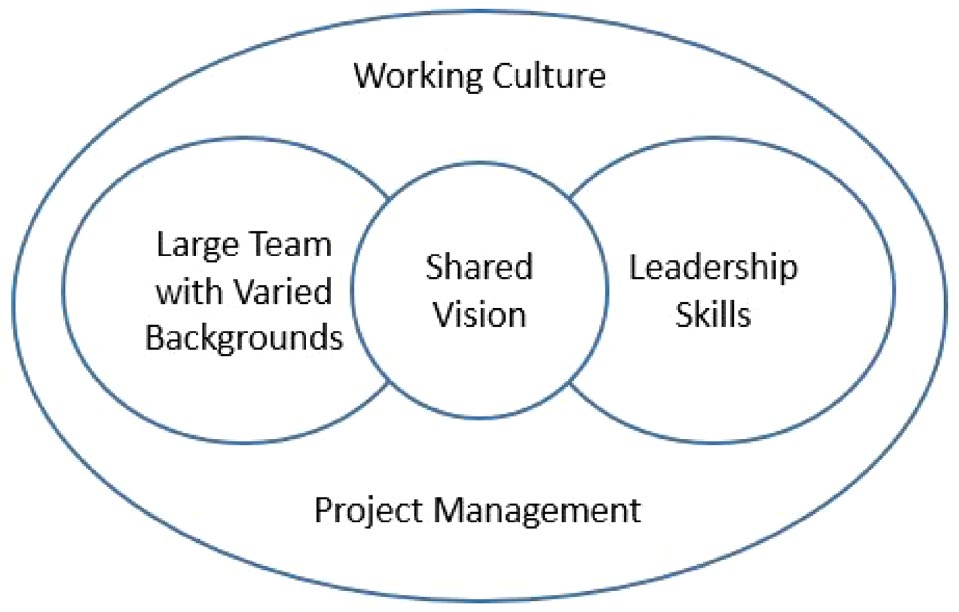
Diagram showing the interaction between factors that contributed to teamwork; a shared vision bridges leadership skills and the large team with varied backgrounds, which is embedded in the working culture and project management

The relationship between the topics discussed in the above sections is shown in Figure 1.

### Access to rapid evaluation methods and digital technology

Successfully implementing rapid evaluation requires access to the right resources that can be summarised as having a large team, using rapid assessment procedure (‘RAP’) sheets to analyse findings iteratively, and providing ongoing immediate feedback to stakeholders.^8^

Our project was entirely virtual due to the COVID-19 lockdown and therefore demanded reliance on technology. We used the synchronous features of MS Teams to share documents, communicate quickly, and keep track of tasks and deadlines. Our team were familiar with MS Teams but it is important to consider time to support any team members who are not familiar with the technology used for any project.

All team members were working remotely, some of whom had caring duties and other work responsibilities, which resulted in a flexible working timetable. Clear and strict deadlines were communicated in advance, and responsibilities were delegated based on skillset and availability as indicated on a live team calendar – a crucial resource for our project.

## Challenges Created by the Rapid Evaluation Process

Team members felt pressured to be on alert for communications and reply quickly, even outside of their normal working hours due to the flexible work schedule. Some data collection tasks were scheduled quickly, which was sometimes too abrupt for some members who could not make those timings. The team leader felt particular pressure as she had to juggle leading this project with other work responsibilities. Finally, this project was ethically approved as a service evaluation and not as research because service evaluation ethics was quicker to attain. This restricted dissemination of this project’s findings and delayed publication. Despite these challenges, team members openly discussed and overcame these challenges because of the culture of trust and support built from the start of the project.

## Conclusion

Our experience illustrates how a rapid yet rigorous evaluation to inform policy can generate actionable results under time pressure that directly informed government policy and practice. This demonstration of how these outcomes can be achieved using entirely online methods suggests that A rapid approach to programme evaluation can be used irrespective of the location of research or public health partners, while also accommodating needs for flexible working. Furthermore, this approach makes the most of short contracts that govern much commissioned research in the UK. We have generated a list of actionable points based on our reflections on the conduct of this project which we hope could be used by anyone planning to conduct a rapid evaluation project to inform policy and practice regardless of the field of study (Table 1).
